# Gene expression-based risk score in diffuse large B-cell lymphoma

**DOI:** 10.18632/oncotarget.807

**Published:** 2012-12-31

**Authors:** Caroline Bret, Bernard Klein, Jérôme Moreaux

**Affiliations:** ^1^ Department of Biological Hematology, St Eloi Hospital, Montpellier, France; ^2^ INSERM U1040, Institute of Research in Biotherapy, Montpellier, France; ^3^ University of Montpellier 1, UFR de Médecine, Montpellier, France

**Keywords:** DLBCL, gene expression profile, risk score, prognosis

## Abstract

Diffuse large B-cell lymphoma (DLBCL) is the most common type of non-Hodgkin lymphoma and displays heterogeneous clinical and molecular characteristics. In this study, high throughput gene expression profiling of DLBCL tumor samples was used to design a 12-gene expression&ndash;based risk score (GERS) predictive for patient's overall survival. GERS allowed identifying a high-risk group comprising 46,4% of the DLBCL patients in two independent cohorts (n=414 and n=69). GERS was shown to be an independent predictor of survival when compared to the previously published prognostic factors, including the International Prognostic Index (IPI). GERS displayed a prognostic value in germinal-center B-cell&ndash;like subgroup (GCB) and activated B cell&ndash;like (ABC) molecular subgroups of patients as well as in DLBCL patients treated with cyclophosphamide, doxorubicin, vincristine and prednisone (CHOP) or Rituximab-CHOP (R-CHOP) regimens. Combination of GERS and IPI lead to a potent prognostic classification of DLBCL patients. Finally, a genomic instability gene signature was highlighted in gene expression profiles of patients belonging to the high-risk GERS-defined group.

## INTRODUCTION

Diffuse large B-cell lymphoma (DLBCL) is the most common type of non-Hodgkin lymphoma, accounting for 30 to 40% of adult non-Hodgkin lymphomas. DLBCL is considered as a heterogeneous disease associated with clinical and biological diversity[1]. Most patients diagnosed with DLBCL achieve long-term remission, but a third of them relapse after conventional Rituximab (R)-based chemotherapy regimens such as combination of cyclophosphamide, doxorubicin, vincristine and prednisone (CHOP)[2].

Prior to therapy, the usual prognostic tool is the International Prognostic Index (IPI), based on clinical and biochemical pre-treatment parameters. In addition to this bio-clinical approach, molecular methods have brought a new definition of DLBCL, demonstrating molecular heterogeneity within morphologically similar tumors and linking gene expression profiles (GEP) to prognosis. Using these approaches, two main subgroups of DLBCL displaying different outcomes after chemotherapy were described: the germinal-center B-cell–like subgroup (GCB) and the activated B cell–like subtype (ABC).

The GCB subgroup is associated with good outcome, accounts for 50% of DLBCL and tumor cells have a healthy germinal-center B cells GEP. ABC subgroup has a poorer outcome, accounts for 30% of cases and tumor cells have a healthy peripheral blood activated B cells GEP, in particular a nuclear factor *k*B (NF-*k*B) signature. The remaining 20% of DLBCL are unclassifiable and associated with the ABC subgroup as “non GCB” forms[3,4]. Using CHOP-like chemotherapy, the 5-year overall survival rates of patients with GCB signature and of patients with ABC profile were 60% and 30% respectively[5].

Based on our previous experience in building powerful risk scores in patients with multiple myeloma[6] or acute myeloid leukemia[7], we aimed to determine a gene expression based-risk score (GERS) in DLBCL patients using publicly-available data. We report the design of GERS using 12 genes whose expression predicts for patients’ overall survival which has strong prognostic value in 2 independent large cohorts of DLBCL patients.

## RESULTS

### Gene Expression-based Risk Score (GERS) in DLBCL

**Figure 1:**
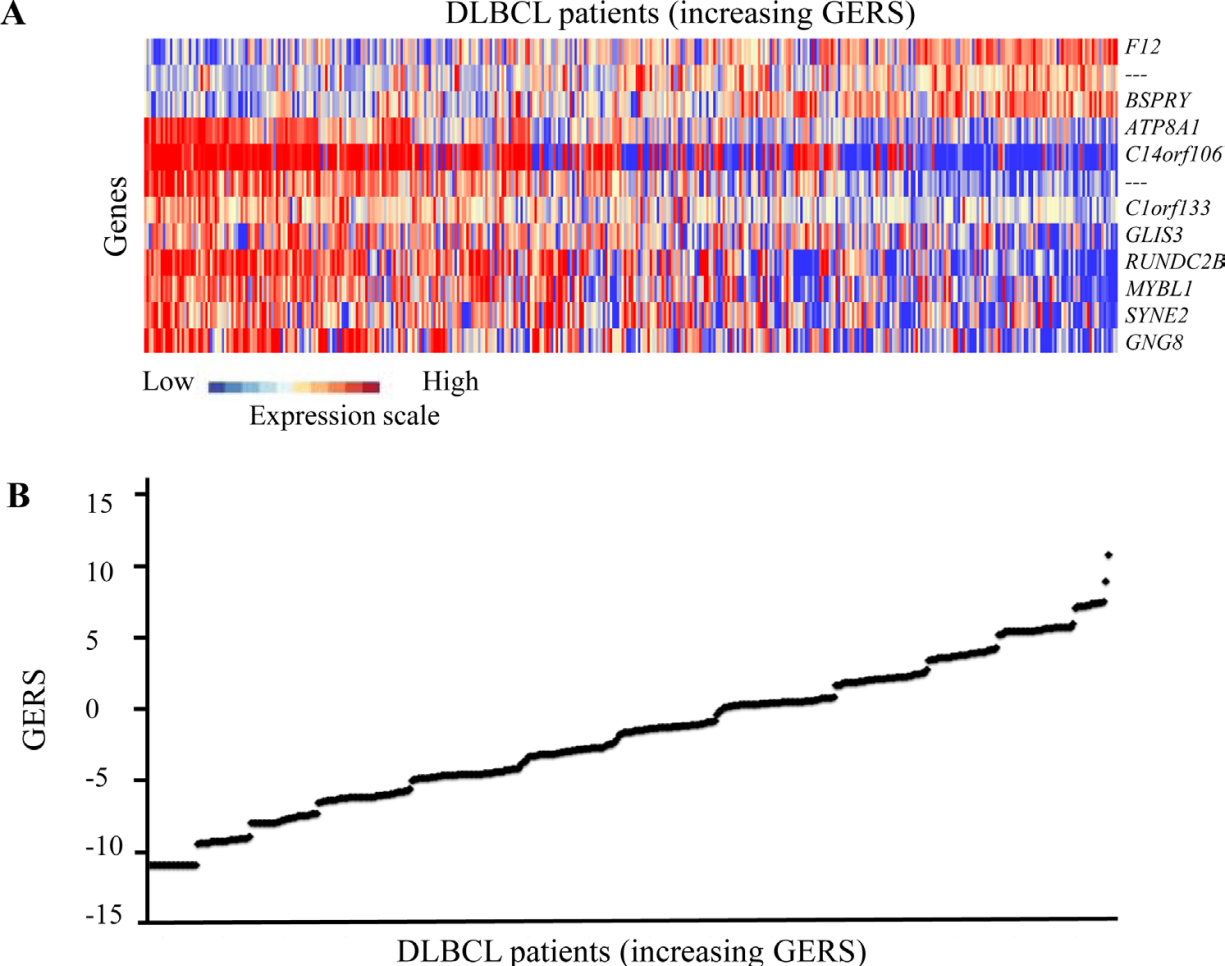
GERS in DLBCL patients A. Clustergram of genes ordered from best to worst prognosis. The level of the probe set signal is displayed from low (deep blue) to high (deep red) expression. B. DLBCL patients (n=414) were ordered by increasing GERS (Gene expression-based risk score).

**Figure 2:**
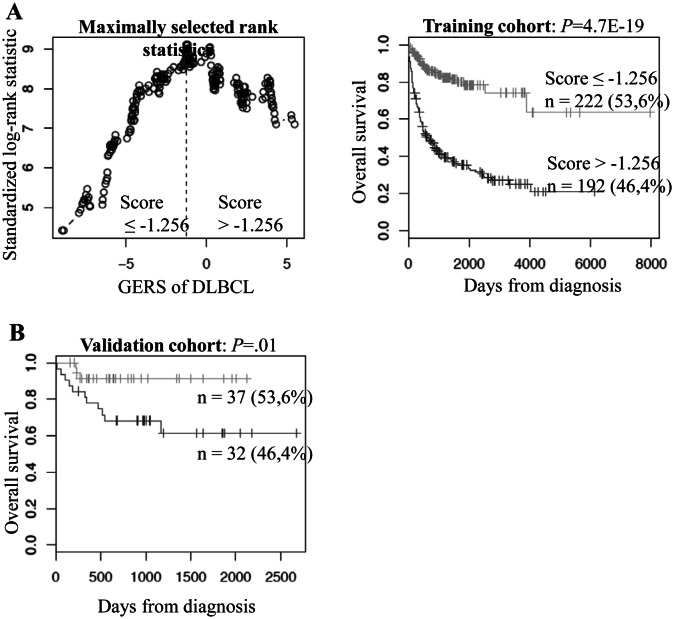
Prognostic value of GERS in DLBCL patients A. Patients of the training cohort (n=414) were ranked according to increasing GERS and a maximum difference in OS was obtained with a score =-1.256, splitting patients into a high risk (46,4%) and a low risk (53,6%) groups. B. The prognostic value of GERS was assayed on an independent cohort of 69 patients (validation cohort). The parameters to compute GERS of patients in the validation cohort and the proportions delineating the 2 prognostic groups were those defined with the training cohort.

**Figure 3:**
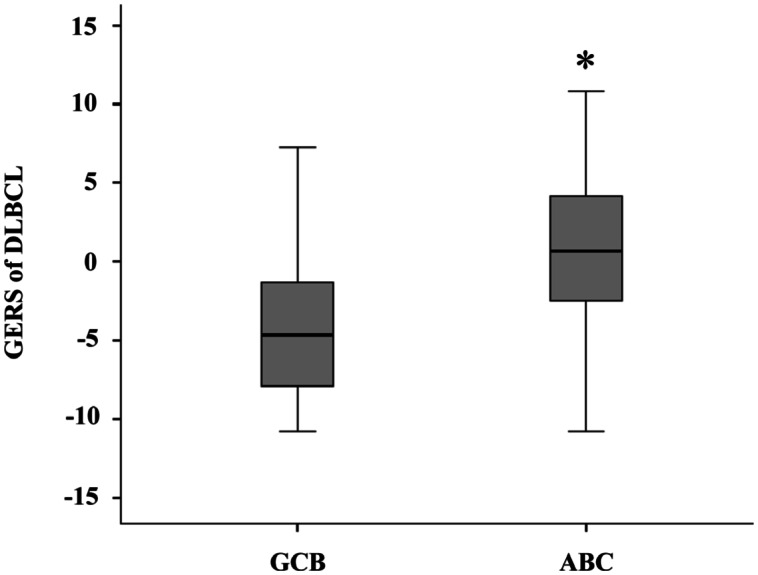
GERS in ABC and GCB molecular subgroups GERS was investigated in activated B-cell like (ABC) and germinal center B-cell like (GCB) molecular subgroups of DLBCL patients (training cohort, n=350). The score was significantly (*) higher in ABC molecular subgroup compared to GCB subgroup (*P*=1.5.10^−28^).

Using Maxstat R function and Benjamini-Hochberg multiple testing correction [8], 12 probe sets were found to have prognostic value for overall survival (adjusted *P* value <.05) in two independent cohorts of patients with newly-diagnosed DLBCL (accession number GSE10846, n=414[9] and accession number GSE23501, n=69[10]) (Table 1). These probe sets probed for 10 unique genes and 2 expressed sequence tag clones. They were used to build the Gene Expression-based Risk Score (GERS). Figures 1A and 1B show expression of the 12 prognostic probe sets and GERS from patients’ tumor samples of the training cohort (ranked according to increasing GERS). When used as a continuous variable, GERS had a prognostic value in the two cohorts of patients with DLBCL (*P*≤10^−4^; data not shown). Patients of the training cohort (n=414) were ranked according to increased prognostic score, and for a given score value X, the difference in survival of patients with a GERS ≤X or >X was computed. A maximum difference in overall survival (OS) was obtained with X=-1.256, splitting patients in a high-risk group (46.4% of patients, GERS >-1.256) with a 22.3 month median OS and a low risk group (53.6% of patients, GERS ≤-1.256) with not reached median survival (Figure 2A). The prognostic value of GERS was validated in an independent DLBCL patient's cohort (n=69) (Figure 2B). With respect to germinal center B-cell like (GCB) and activated B-cell like (ABC) molecular subgroups[4], GERS was significantly higher (*P*=1.5.10^−28^) in ABC molecular subgroup compared to GCB subgroup (Figure 3).

**Table 1:** List of the 12 probe sets associated with prognostic value in DLBCL patients Hazard ratios (HR) are indicated for each gene used to design GEP-based risk score (GERS) in DLBCL patients. Probe sets are sorted by increasing HR.

Probe set	Gene symbol	Gene name	Hazard Ratio (HR)
1569773_at	ATP8A1	ATPase, aminophospholipid transporter (APLT), class I, type 8A, member 1	0.2353597
229435_at	GLIS3	GLIS family zinc finger 3	0.3969455
1554413_s_at	RUNDC2B	RUN domain containing 2B	0.4002619
230121_at	C1orf133	Chromosome 1 open reading frame 133	0.4067606
240777_at	SYNE2	Spectrin repeat containing, nuclear envelope 2	0.4144356
213906_at	MYBL1	v-myb myeloblastosis viral oncogene homolog (avian)-like 1	0.4203686
235743_at	---	---	0.4269757
234284_at	GNG8	Guanine nucleotide binding protein (G protein), gamma 8	0.4384588
206500_s_at	C14orf106	Chromosome 14 open reading frame 106	0.4767180
218792_s_at	BSPRY	B-box and SPRY domain containing	2.0686670
221275_s_at	---	---	2.1554317
205774_at	F12	Coagulation factor XII (Hageman factor)	2.6380675

Cox analysis was used to determine whether the GERS provides additional prognostic information compared to previously-identified poor outcome-related factors such as GCB or ABC molecular subgroups and the IPI (low risk group/IPI score 0 or 1, low-intermediate risk group/IPI score 2, high-intermediate risk group/IPI score 3 and high risk group/IPI score 4 or 5). Using univariate analyses, GERS, age, ABC/GCB molecular subgroups and IPI had prognostic value (*P*<.0001, Table 2A). When compared two by two, GERS tested with age, GCB-ABC molecular subgroups or IPI remained significant (*P*<.0001, *P*=.03 and *P*<.0001 respectively, Table 2B). When all parameters were tested together, only GERS and IPI kept prognostic values (Table 2C).

**Table 2:** Cox univariate and multivariate analysis of OS in DLBCL patient's training cohort (n=414) The prognostic factors were tested as single variable (A) or multivariables (B, C) using Cox-model. P-values and the hazard ratios (HR) are shown. NS: not significant at a 5% threshold.

A.	Overall survival (n=414)	
Prognostic variable	HR	P value
GERS	4.62	<.0001
Age (>60 years)	2.2	<.0001
GCB-ABC molecular subgroups	2.75	<.0001
IPI	1.79	<.0001

B.	Overall survival (n=414)	
Prognostic variables compared two by two	HR	P value
GERS	4.25	<.0001
Age (>60 years)	1.82	<.0001
GERS	3.75	<.0001
GCB-ABC molecular subgroups	1.51	.03
GERS	4.60	<.0001
IPI	1.83	<.0001

C.	Overall survival (n=414)	
All prognostic variables	HR	P value
GERS	4.11	<.0001
Age (>60 years)	1.26	NS
GCB-ABC molecular subgroups	1.27	NS
IPI	1.74	<.0001

**Figure 4:**
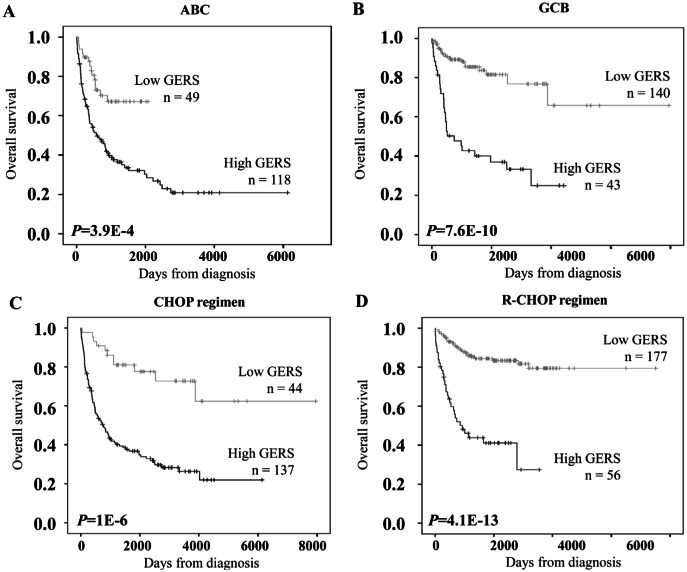
Prognostic prediction applying GERS in ABC/GCB subgroups of DLBCL patients The prognostic value of GERS was tested in the training cohort for DLBCL patients of ABC molecular subgroup (A, n=167), GCB molecular subgroup (B, n=183), treated by CHOP regimen (C, n=181) or by R-CHOP regimen (D, n=233).

Interestingly, GERS had prognostic value in GCB or ABC molecular subgroups. GERS segregated patients of ABC subgroup into a high-risk group with 19.1 month median OS and a low risk group with not reached median OS (*P*=4.9E-4, Figure 4A). GERS separated patients of GCB subgroup into a high-risk group with 24.6 month median OS and a low risk group with not reached median OS (*P*=7.6E-10, Figure 4B). Of interest, GERS remained a powerful prognostic factor separating DLBCL patients treated with CHOP regimen or R-CHOP regimen (*P*= 1E-6 and *P*= 4.1E-13 respectively, Figures 4C and 4D).

### Combining prognostic information of GERS and IPI into a single staging

**Figure 5:**
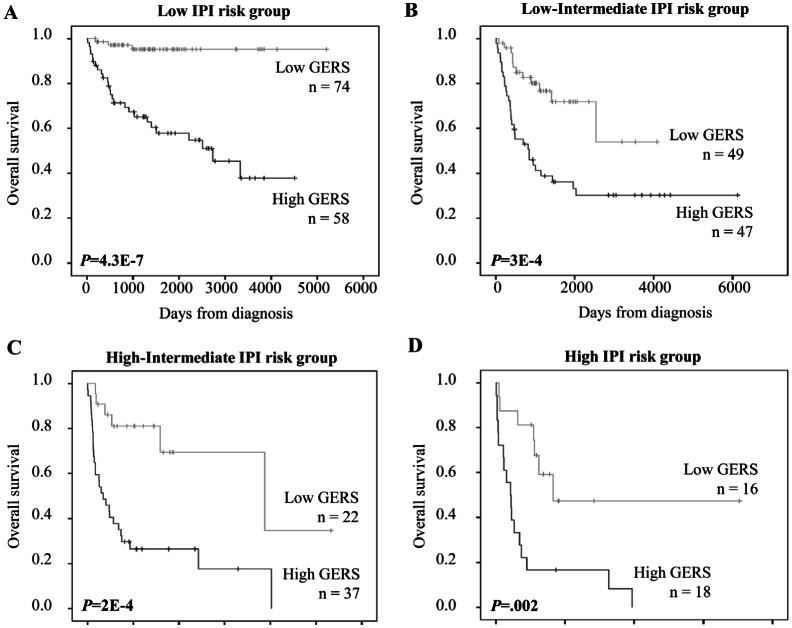
Prognostic value of GERS for subgroups of DLBCL patients defined by international prognostic index (IPI) DLBCL patients within low, low-intermediate, high-intermediate or high-risk IPI groups were split using GERS cutoff (-1.256). IPI was available for 321 of the 414 patients of the training cohort.

Since GERS and IPI displayed independent prognostic information, we found that GERS allowed splitting patients with low risk IPI group into a high-risk group with 89.9 month median OS and a low risk group of patients with not reached median survival (*P*=4.3E-7, Figure 5A). The same holds true for patients within low-intermediate risk IPI group (segregated in a high-risk group with a 27.7 month median OS and a low risk group with not reached median survival, P=3E-4, Figure 5B), for patients within high-intermediate risk IPI group (separated into a high-risk group with 11.3 month median OS and a low risk group with 54.9 month median OS, P=2E-4, Figure 5C) and for patients within high risk IPI group (split into a high-risk group with 6.9 month median OS and a low risk group with 27.1 month median OS, *P*=.002, Figure 5D). To combine the prognostic information of GERS and IPI, a staging was built, scoring patients from 1 to 8 (2 GERS sub-groups in each of the 4 IPI groups as previously described).

**Figure 6:**
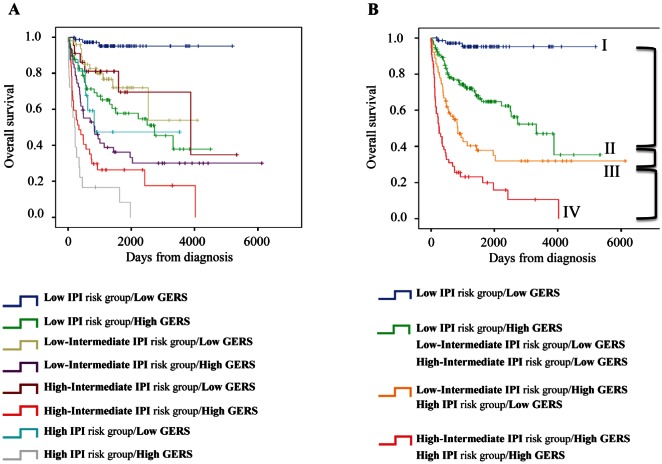
Combination of the prognostic information of GERS and IPI A. Kaplan-Meier analyses were performed to combine the prognostic information of GERS and IPI risk classifications. Patients were scored from 1 to 8 (2 GERS sub-groups in each of the 4 IPI groups). B. After merging consecutive groups with no prognostic difference, 4 patient groups with different overall survival (OS) were obtained: I, II, III, IV (patients of the training cohort, n=321).

Kaplan-Meier analysis with the 8 patient groups of the training cohort was performed (Figure 6A). When 2 consecutive groups showed no prognostic difference, they were merged yielding to 4 patient groups with different OS (Figure 6B). Group I comprised 23% of patients with low IPI risk/low GERS. Group II accounted for 40.2% of patients with low IPI risk/high GERS, low-intermediate IPI risk/low GERS and high-intermediate IPI risk/low GERS. Group III comprised 19.6% of patients with low IPI risk/high GERS and high IPI risk/low GERS. Group IV accounted for 17.2% of patients with low-intermediate IPI risk/high GERS and high IPI risk/high GERS. Group I patients had a not reached median OS, patients of groups II, III and IV had respectively a median OS of 109.3 months, 27.7 months and 8.5 months (Figure 6B).

### Tumor cells of patients in GERS high-risk group have a genomic instability gene signature

Gene set enrichment analysis was performed comparing gene expression profiles of DLBCL patients with high and low GERS (n=192 and n=222 respectively in the training cohort). Genes related with genomic instability pathways (gene sets: RESPONSE_TO_UV and RESPONSE_TO_RADIATION, *P*<.001, supplementary Figure S1 and supplementary Tables S1 and S2) and apoptosis (gene set: MITOCHONDRIAL_OUTER_MEMBRANE, *P*<.001, supplementary Figure S1 and supplementary Tables S3) were enriched in GERS high risk group. Conversely, gene encoding for protein translation (gene set: STRUCTURAL_CONSTITUENT_OF_RIBOSOME, *P*=.04, supplementary Figure S2 and supplementary Tables S4) were enriched in GERS low risk group.

## DISCUSSION

Given the genetic heterogeneity of hematological malignancies, GEP of tumor cells have enabled the identification of additional molecular heterogeneity associated with prognostic value[7,11-20]. DLBCL is characterized by its biological heterogeneity leading to heterogeneous responses to therapy and different survival outcomes. Using GEP, several DLBCL subgroups with different OS were identified mainly based on the cell origin or the tumor microenvironment, including the ABC and GCB subtypes[4] and the stromal signatures[9]. Various prognostic models have been developed to stratify risk in patients with newly diagnosed DLBCL.

Using publicly data from two independent patients’ cohort[9,10], a GEP-based risk score (GERS) was built, incorporating prognostic information of 12 genes/expressed sequence tag clones in DLBCL patients. GERS first allowed splitting DLBCL patients of the 2 independent cohorts into a high risk and a low risk groups. GERS was shown to be an independent predictor for OS when compared to the previously published prognostic factors. Interestingly, when combined to IPI, GERS led to a more potent prognostic classification of DLBCL patients.

Besides the powerful prognostic value of GERS, the current study highlights pathways that could be involved in poor prognostic DLBCL. Among the 12 prognostic genes used to build GERS, the *BSPRY* gene encodes B-box and SPRY domain containing protein. A high expression of BSPRY in tumor cells was associated with poor OS in DLBCL patients according to GERS. In murine models, *BSPRY* gene shows an ubiquitous expression in various tissues, the highest expression being found in testis with two alternative splice isoforms (BSPRY-1 and BSPRY-2)[21]. BSPRY protein can interact with 14-3-3 proteins[22] and is involved in the regulation of epithelial Ca^2+^ transport *via* the modulation of Transient Receptor Potential Vallinoid 5 (TRPV5) activity[23]. More recently, the function of the two alternative splice isoforms was investigated in embryonic stem (ES) cells and early embryonic development. Interestingly, the knockdown of *BSPRY-1 and BSPRY-2* resulted in ES cells differentiation and in developmental retardation of early embryos *in vitro*[21]. These data emphasize an implication of BSPRY in ES cell pluripotency and early embryonic development. The involvement of BSPRY in cancer stem cells biology has not been explored. Taken together, these data suggest that BSPRY could be involved in B lymphomagenesis. Two other genes -*ATP8A1* and *MYBL1*- used to build GERS could be of interest. Their low expression in tumor cells was associated with poor OS. *ATP8A1* encodes for the ATPase aminophospholipid transporter class I type 8A member 1, which belongs to the family of aminophospholipid translocases. ATP8A1 is involved in the translocation of amphipaths such as phosphatidylserine (PS) and phosphatidylethanolamine (PE) within the plasma membrane, which can occur during apoptosis[24,25]. ATP8A1 is implicated in the exposure of PS in the outer leaflet of the plasma membrane of neuroblastoma cells, this alteration of surface lipid components leading to phagocytosis of cancer cells[26]. *MYBL1,* also known as *A-myb,* encodes for v-myb myeloblastosis viral oncogene homolog (avian)-like 1, a transcription factor. *MYBL1* belongs to the MYB family, including the v-myb oncogene and the *C-myb* and *B-myb* genes[27,28]. In human hematopoietic cells, MYBL1 is specifically expressed by centroblasts[29]. MYBL1 is a survival factor for murine B lymphomas transactivating c-myc expression[30] and is overexpressed in human acute and chronic B-cell neoplasias[31]. In transgenic mice, ectopic expression of MYBL1 induces lymphoid hyperplasia in lymph nodes with an expansion of follicular center B cells[32]. Interestingly, the previously published GCB signature also included the gene encoding *MYBL1*[9] as well as the signature published by the group of A. Alizadeh[4]. MYBL1 could be involved in DLBCL pathogenesis in addition to its role in Burkitt lymphoma or chronic lymphoid leukemia (CLL)[31].

Interestingly, GSEA analysis highlighted a significant enrichment of genes associated with genomic instability and apoptosis in tumor cells of patients within high risk GERS group (supplementary Figure S1 and supplementary Tables S1, S2 and S3). In particular, enrichment for genes encoding for nucleotide excision DNA repair (NER) pathway (genes belonging to the ERCC family: *ERCC2/XPD*, *ERCC3/XPB*, *ERCC4/XPF* and *ERCC8/CSA*) and an overexpression of *MCL1* were obtained. Transgenic mice expressing an *MCL1* transgene in lymphoid tissues develop lymphoma after a long latency[33]. In non-Hodgkin lymphoma, *MCL1* expression was significantly lower in patients in complete remission than with progressive disease[34]. These data suggest that targeting NER DNA repair or *MCL1* could have a therapeutic interest in patients with a high risk GERS. F11782, a novel dual catalytic inhibitor of topoisomerases I and II, is a potent inhibitor of NER[35]. More recently, it was demonstrated that PARP activation following UV radiation exposure promoted association between PARP-1 and XPA, a central protein in NER. Administration of PARP inhibitors confirmed that poly-(ADP-ribose) mediated PARP-1 association with XPA and decreased UV radiation-stimulated XPA chromatin association. These observations illustrate the function of PARP in NER DNA repair[36]. Clinical grade PARP inhibitors, alone or in combination with chemotherapy, could be of clinical interest in the high-risk group of DLBCL patients identified with GERS. In DLBCL tumors with low risk GERS, GSEA analysis highlighted an enrichment of genes encoding for protein translational machinery (supplementary Figure S2 and supplementary Tables S4). Deregulated protein synthesis plays an important role in human cancer and deregulated translational control has been recognized as an integral part of the malignant state [37-39]. Multiple drugs have been developed to target molecules involved in the regulation of protein translation. Rapamycin and rapalogs (temsirolimus, everolimus, and deferolimus) inhibit mTORC1 signaling[40-42]. Other small molecule inhibitors (Torin1, PP242 and PP30) have been developed to target the mTOR kinase domain, which may inhibit mTORC1 and mTORC2 signaling pathways[43] [44]. CGP57380 has been developed as an ATP competitive inhibitor of the MNK kinases, which may prevent a subsequent round of translation on the same mRNA[45] [46]. Other drugs have been found to block the recruitment of eIF4E to the eIF4F ternary complex, including 4EGI-1 and Ribavirin. They inhibit both translation initiation and eIF4E-mediated transport of mRNA[47-50]. These inhibitors may constitute a potential therapeutic approach in these subgroups of DLBCL patients.

Given the heterogeneity of DLBCL patients, the current GERS combined with IPI could help identifying high-risk patients who may benefit from intensive therapeutic strategies and new targeted treatments.

## MATERIALS AND METHODS

### Patients

Gene expression microarray data from two independent cohorts of patients diagnosed with DLBCL were used. The first cohort, used as the training cohort, comprised 414 patients[9] and the second one as the validation cohort comprised 69 patients [10]. Pre-treatment clinical characteristics of patients were previously published by the groups of G. Lenz and of R. Shaknovich. Affymetrix gene expression data are publicly available *via* the online Gene Expression Omnibus (http://www.ncbi.nlm.nih.gov/geo/) under accession number GSE10846 and GSE23501. They were performed using Affymetrix HG-U133 plus 2.0 microarrays for the two cohorts of patients. The data were analyzed with Microarray Suite version 5.0 (MAS 5.0), using Affymetrix default analysis settings and global scaling as normalization method. The trimmed mean target intensity of each array was arbitrarily set to 500.

### Gene expression profiling and statistical analyses

The statistical significance of differences in overall survival between groups of patients was calculated by the log-rank test. Multivariate analysis was performed using the Cox proportional hazards model. Survival curves were plotted using the Kaplan-Meier method. All these analyses have been done with R.2.10.1 and bioconductor version 2.5. Gene annotation and networks were generated through the use of Ingenuity Pathways Analysis (Ingenuity_®_ Systems, Redwood City, CA).

### Selection of prognostic genes on the training set (cohort of 414 patients)

Probe sets were selected for prognostic significance using Maxstat R function and Benjamini Hochberg multiple testing correction[8], yielding 12 significant probe sets in the two independent cohorts of patients with DLBCL (Table 1).

### Building the gene expression-based risk score (GERS)

To gather prognostic information of the 12 prognostic probe sets within one parameter, the GERS of DLBCL was built as the sum of the beta coefficients weighted by ± 1 according to the patient signal above or below the probe set Maxstat value[8].

### Validation in the independent cohort of patients

The GERS of DLBCL patients was individually calculated and patients were grouped according to the prognostic models and cut-offs from the training cohort. The prognostic value of this scoring was evaluated using log-rank statistics and Cox models.

### Gene set enrichment analysis (GSEA)

We compared the gene expression levels from high risk GERS versus low risk GERS DLBCL patients and picked up the genes which had significant different expression for Gene set enrichment analysis (GSEA). Gene set enrichment analysis was carried out by computing overlaps with canonical pathways and gene ontology gene sets obtained from the Broad Institute[51].

## Supplementary Figures and Tables





## References

[1] Staudt LM, Dave S (2005). The biology of human lymphoid malignancies revealed by gene expression profiling. Adv. Immunol.

[2] Siegel R, Naishadham D, Jemal A (2012). Cancer statistics, 2012. CA Cancer J Clin.

[3] Rosenwald A, Wright G, Chan WC, Connors JM, Campo E, Fisher RI, Gascoyne RD, Muller-Hermelink HK, Smeland EB, Giltnane JM, Hurt EM, Zhao H, Averett L, Yang L, Wilson WH, Jaffe ES, Simon R, Klausner RD, Powell J, Duffey PL, Longo DL, Greiner TC, Weisenburger DD, Sanger WG, Dave BJ, Lynch JC, Vose J, Armitage JO, Montserrat E, López-Guillermo A, Grogan TM, Miller TP, LeBlanc M, Ott G, Kvaloy S, Delabie J, Holte H, Krajci P, Stokke T, Staudt LM (2002). Lymphoma/Leukemia Molecular Profiling Project. The use of molecular profiling to predict survival after chemotherapy for diffuse large-B-cell lymphoma. N. Engl. J. Med.

[4] Alizadeh AA, Eisen MB, Davis RE, Ma C, Lossos IS, Rosenwald A, Boldrick JC, Sabet H, Tran T, Yu X, Powell JI, Yang L, Marti GE, Moore T, Hudson J, Lu L, Lewis DB, Tibshirani R, Sherlock G, Chan WC, Greiner TC, Weisenburger DD, Armitage JO, Warnke R, Levy R, Wilson W, Grever MR, Byrd JC, Botstein D, Brown PO, Staudt LM (2000). Distinct types of diffuse large B-cell lymphoma identified by gene expression profiling. Nature.

[5] Wright G, Tan B, Rosenwald A, Hurt EH, Wiestner A, Staudt LM (2003). A gene expression-based method to diagnose clinically distinct subgroups of diffuse large B cell lymphoma. Proc. Natl. Acad. Sci. U.S.A.

[6] Moreaux J, Klein B, Bataille R, Descamps G, Maïga S, Hose D, Goldschmidt H, Jauch A, Rème T, Jourdan M, Amiot M, Pellat-Deceunynck C (2011). A high-risk signature for patients with multiple myeloma established from the molecular classification of human myeloma cell lines. Haematologica.

[7] Samra EB, Klein B, Commes T, Moreaux J (2012). Development of gene expression-based risk score in cytogenetically normal acute myeloid leukemia patients. Oncotarget.

[8] Kassambara A, Hose D, Moreaux J, Walker BA, Protopopov A, Rème T, Pellestor F, Pantesco V, Jauch A, Morgan G, Goldschmidt H, Klein B (2012). Genes with a spike expression are clustered in chromosome (sub)bands and spike (sub)bands have a powerful prognostic value in patients with multiple myeloma. Haematologica.

[9] Lenz G, Wright G, Dave SS, Xiao W, Powell J, Zhao H, Xu W, Tan B, Goldschmidt N, Iqbal J, Vose J, Bast M, Fu K, Weisenburger DD, Greiner TC, Armitage JO, Kyle A, May L, Gascoyne RD, Connors JM, Troen G, Holte H, Kvaloy S, Dierickx D, Verhoef G, Delabie J, Smeland EB, Jares P, Martinez A, Lopez-Guillermo A, Montserrat E, Campo E, Braziel RM, Miller TP, Rimsza LM, Cook JR, Pohlman B, Sweetenham J, Tubbs RR, Fisher RI, Hartmann E, Rosenwald A, Ott G, Muller-Hermelink HK, Wrench D, Lister TA, Jaffe ES, Wilson WH, Chan WC, Staudt LM (2008). Lymphoma/Leukemia Molecular Profiling Project. Stromal gene signatures in large-B-cell lymphomas. N. Engl. J. Med.

[10] Shaknovich R, Geng H, Johnson NA, Tsikitas L, Cerchietti L, Greally JM, Gascoyne RD, Elemento O, Melnick A (2010). DNA methylation signatures define molecular subtypes of diffuse large B-cell lymphoma. Blood.

[11] Moreaux J, Cremer FW, Rème T, Raab M, Mahtouk K, Kaukel P, Pantesco V, De Vos J, Jourdan E, Jauch A, Legouffe E, Moos M, Fiol G, Goldschmidt H, Rossi JF, Hose D, Klein B (2005). The level of TACI gene expression in myeloma cells is associated with a signature of microenvironment dependence versus a plasmablastic signature. Blood.

[12] Moreaux J, Hose D, Rème T, Jourdan E, Hundemer M, Legouffe E, Moine P, Bourin P, Moos M, Corre J, Möhler T, De Vos J, Rossi JF, Goldschmidt H, Klein B (2006). CD200 is a new prognostic factor in multiple myeloma. Blood.

[13] Condomines M, Hose D, Raynaud P, Hundemer M, De Vos J, Baudard M, Moehler T, Pantesco V, Moos M, Schved J-F, Rossi JF, Rème T, Goldschmidt H, Klein B (2007). Cancer/testis genes in multiple myeloma: expression patterns and prognosis value determined by microarray analysis. J. Immunol.

[14] Bret C, Hose D, Rème T, Sprynski A-C, Mahtouk K, Schved J-F, Quittet P, Rossi JF, Goldschmidt H, Klein B (2009). Expression of genes encoding for proteins involved in heparan sulphate and chondroitin sulphate chain synthesis and modification in normal and malignant plasma cells. British Journal of Haematology.

[15] Bou Samra E, Moreaux J, Vacheret F, Mills K, Rufflé F, Chiesa J, Piquemal D, Boureux A, Lavabre-Bertrand T, Jourdan E, Commes T (2012). New prognostic markers, determined using gene expression analyses, reveal two distinct subtypes of chronic myelomonocytic leukaemia patients. British Journal of Haematology.

[16] Frick M, Dörken B, Lenz G (2012). New insights into the biology of molecular subtypes of diffuse large B-cell lymphoma and Burkitt lymphoma. Best Practice & Research Clinical Haematology. Elsevier Ltd.

[17] Hendrix A, Braems G, Bracke M, Seabra M, Gahl W, De Wever O, Westbroek W (2010). The secretory small GTPase Rab27B as a marker for breast cancer progression. Oncotarget.

[18] Castellano G, Torrisi E, Ligresti G, Nicoletti F, Malaponte G, Traval S, McCubrey JA, Canevari S, Libra M (2010). Yin Yang 1 overexpression in diffuse large B-cell lymphoma is associated with B-cell transformation and tumor progression. Cell Cycle.

[19] Higgins GS, Harris AL, Prevo R, Helleday T, McKenna WG, Buffa FM (2010). Overexpression of POLQ confers a poor prognosis in early breast cancer patients. Oncotarget.

[20] Ertel A, Dean JL, Rui H, Liu C, Witkiewicz A, Knudsen KE, Knudsen ES (2010). RB-pathway disruption in breast cancer: Differential association with disease subtypes, disease-specific prognosis and therapeutic response. Cell Cycle.

[21] Ikeda M, Inoue F, Ohkoshi K, Yokoyama S, Tatemizo A, Tokunaga T, Furusawa T (2012). B-box and SPRY Domain Containing Protein (BSPRY) is Associated with the Maintenance of Mouse Embryonic Stem Cell Pluripotency and Early Embryonic Development. J. Reprod. Dev.

[22] Birkenfeld J, Kartmann B, Anliker B, Ono K, Schlötcke B, Betz H, Roth D (2003). Characterization of zetin 1/rBSPRY, a novel binding partner of 14-3-3 proteins. Biochem. Biophys. Res. Commun.

[23] van de Graaf SFJ, van der Kemp AWCM, van den Berg D, van Oorschot M, Hoenderop JGJ, Bindels RJM (2006). Identification of BSPRY as a novel auxiliary protein inhibiting TRPV5 activity. J. Am. Soc. Nephrol.

[24] Fadok VA, Voelker DR, Campbell PA, Cohen JJ, Bratton DL, Henson PM (1992). Exposure of phosphatidylserine on the surface of apoptotic lymphocytes triggers specific recognition and removal by macrophages. J. Immunol.

[25] Ikeda M, Kihara A, Igarashi Y (2006). Lipid asymmetry of the eukaryotic plasma membrane: functions and related enzymes. Biol. Pharm. Bull.

[26] Levano K, Sobocki T, Jayman F, Debata PR, Sobocka MB, Banerjee P (2009). A genetic strategy involving a glycosyltransferase promoter and a lipid translocating enzyme to eliminate cancer cells. Glycoconj. J.

[27] Lyon J, Robinson C, Watson R (1994). The role of Myb proteins in normal and neoplastic cell proliferation. Crit Rev Oncog.

[28] Introna M, Luchetti M, Castellano M, Arsura M, Golay J (1994). The myb oncogene family of transcription factors: potent regulators of hematopoietic cell proliferation and differentiation. Semin. Cancer Biol.

[29] Golay J, Broccoli V, Lamorte G, Bifulco C, Parravicini C, Pizzey A, Thomas NS, Delia D, Ferrauti P, Vitolo D, Introna M (1998). The A-Myb transcription factor is a marker of centroblasts in vivo. J. Immunol.

[30] Arsura M, Hofmann CS, Golay J, Introna M, Sonenshein GE (2000). A-myb rescues murine B-cell lymphomas from IgM-receptor-mediated apoptosis through c-myc transcriptional regulation. Blood.

[31] Golay J, Luppi M, Songia S, Palvarini C, Lombardi L, Aiello A, Delia D, Lam K, Crawford DH, Biondi A, Barbui T, Rambaldi A, Introna M (1996). Expression of A-myb, but not c-myb and B-myb, is restricted to Burkitt's lymphoma, sIg+ B-acute lymphoblastic leukemia, and a subset of chronic lymphocytic leukemias. Blood.

[32] DeRocco SE, Iozzo R, Ma XP, Schwarting R, Peterson D, Calabretta B (1997). Ectopic expression of A-myb in transgenic mice causes follicular hyperplasia and enhanced B lymphocyte proliferation. Proc. Natl. Acad. Sci. U.S.A.

[33] Zhou P, Levy NB, Xie H, Qian L, Lee CY, Gascoyne RD, Craig RW (2001). MCL1 transgenic mice exhibit a high incidence of B-cell lymphoma manifested as a spectrum of histologic subtypes. Blood.

[34] Kuramoto K, Sakai A, Shigemasa K, Takimoto Y, Asaoku H, Tsujimoto T, Oda K, Kimura A, Uesaka T, Watanabe H, Katoh O (2002). High expression of MCL1 gene related to vascular endothelial growth factor is associated with poor outcome in non-Hodgkin's lymphoma. British Journal of Haematology.

[35] Barret JM, Cadou M, Hill BT (2002). Inhibition of nucleotide excision repair and sensitisation of cells to DNA cross-linking anticancer drugs by F 11782, a novel fluorinated epipodophylloid. Biochem. Pharmacol.

[36] King BS, Cooper KL, Liu KJ, Hudson LG (2012). Poly(ADP-ribose) Contributes to an Association between Poly(ADP-ribose) Polymerase-1 and Xeroderma Pigmentosum Complementation Group A in Nucleotide Excision Repair. J. Biol. Chem.

[37] Ruggero D, Pandolfi PP (2003). Does the ribosome translate cancer?. Nat. Rev. Cancer.

[38] Hagner PR, Schneider A, Gartenhaus RB (2010). Targeting the translational machinery as a novel treatment strategy for hematologic malignancies. Blood.

[39] Thomas G, Hall MN (1997). TOR signalling and control of cell growth. Curr. Opin. Cell Biol.

[40] Guertin DA, Sabatini DM (2007). Defining the role of mTOR in cancer. Cancer Cell.

[41] Blagosklonny MV (2012). Rapalogs in cancer prevention: Anti-aging or anticancer?. Cancer Biol Ther.

[42] Anisimov VN, Zabezhinski MA, Popovich IG, Piskunova TS, Semenchenko AV, Tyndyk ML, Yurova MN, Antoch MP, Blagosklonny MV (2010). Rapamycin Extends Maximal Lifespan in Cancer-Prone Mice. The American Journal of Pathology. American Society for Investigative Pathology.

[43] Thoreen CC, Kang SA, Chang JW, Liu Q, Zhang J, Gao Y, Reichling LJ, Sim T, Sabatini DM, Gray NS (2009). An ATP-competitive mammalian target of rapamycin inhibitor reveals rapamycin-resistant functions of mTORC1. J. Biol. Chem.

[44] Feldman ME, Apsel B, Uotila A, Loewith R, Knight ZA, Ruggero D, Shokat KM (2009). Active-site inhibitors of mTOR target rapamycin-resistant outputs of mTORC1 and mTORC2. PLoS Biol.

[45] Bianchini A, Loiarro M, Bielli P, Busà R, Paronetto MP, Loreni F, Geremia R, Sette C (2008). Phosphorylation of eIF4E by MNKs supports protein synthesis, cell cycle progression and proliferation in prostate cancer cells. Carcinogenesis.

[46] Phillips A, Blaydes JP (2008). MNK1 and EIF4E are downstream effectors of MEKs in the regulation of the nuclear export of HDM2 mRNA. Oncogene.

[47] Kentsis A, Topisirovic I, Culjkovic B, Shao L, Borden KLB (2004). Ribavirin suppresses eIF4E-mediated oncogenic transformation by physical mimicry of the 7-methyl guanosine mRNA cap. Proc. Natl. Acad. Sci. U.S.A.

[48] Assouline S, Culjkovic B, Cocolakis E, Rousseau C, Beslu N, Amri A, Caplan S, Leber B, Roy D-C, Miller WH, Borden KLB (2009). Molecular targeting of the oncogene eIF4E in acute myeloid leukemia (AML): a proof-of-principle clinical trial with ribavirin. Blood.

[49] Moerke NJ, Aktas H, Chen H, Cantel S, Reibarkh MY, Fahmy A, Gross JD, Degterev A, Yuan J, Chorev M, Halperin JA, Wagner G (2007). Small-molecule inhibition of the interaction between the translation initiation factors eIF4E and eIF4G. Cell.

[50] Tamburini J, Green AS, Bardet V, Chapuis N, Park S, Willems L, Uzunov M, Ifrah N, Dreyfus F, Lacombe C, Mayeux P, Bouscary D (2009). Protein synthesis is resistant to rapamycin and constitutes a promising therapeutic target in acute myeloid leukemia. Blood.

[51] Subramanian A, Tamayo P, Mootha VK, Mukherjee S, Ebert BL, Gillette MA, Paulovich A, Pomeroy SL, Golub TR, Lander ES, Mesirov JP (2005). Gene set enrichment analysis: a knowledge-based approach for interpreting genome-wide expression profiles. Proc. Natl. Acad. Sci. U.S.A.

