# Supplementary material for: Gene expression-based risk score in diffuse large B-cell lymphoma
Source: Oncotarget. 2012 Dec 31;3(12):1700–10. doi: 10.18632/oncotarget.807 (PMC3681505; doi:10.18632/oncotarget.807)
Supplement: Supplementary file 1 [file oncotarget-03-1700-s001.pdf]

Gene expression-based risk score in diffuse large B-cell lymphoma - Bret et al

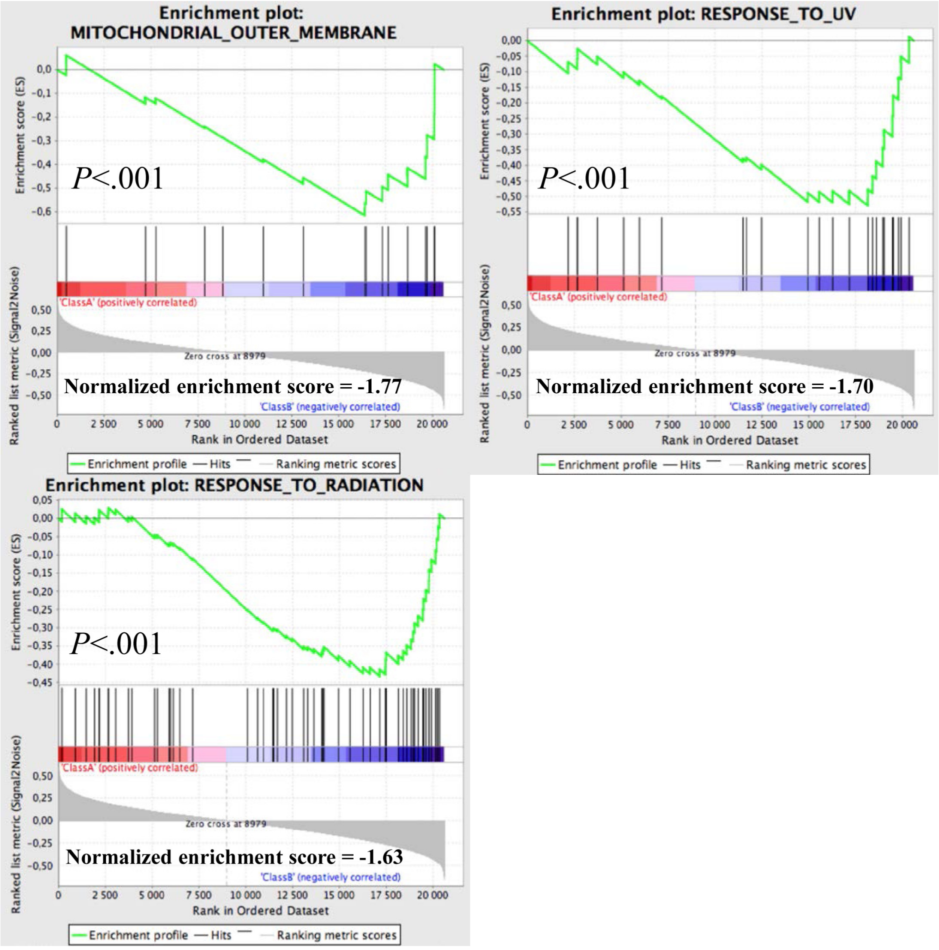

Supplementary Figure 1: Top gene sets significantly associated with high GERS group. GSEA enrichment plots with the absolute enrichment p value and the normalized enrichment score of the gene set.

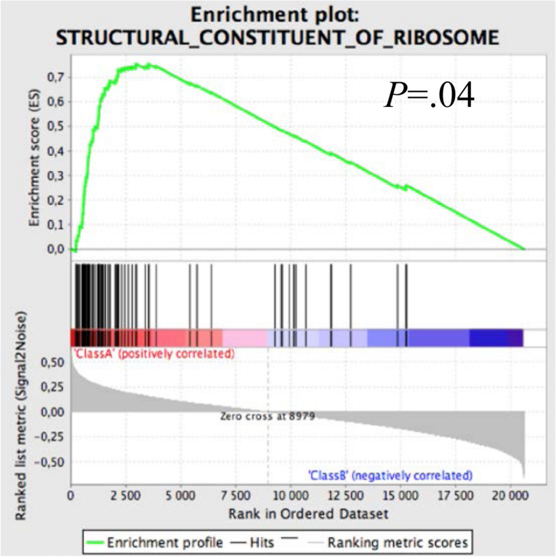

Supplementary Figure 2: Top gene set significantly associated with low GERS group. GSEA enrichment plot with the absolute enrichment p value and the normalized enrichment score of the gene set.
